# Collaborative knotworking – transforming clinical teaching practice through faculty development

**DOI:** 10.1186/s12909-020-02407-8

**Published:** 2020-12-09

**Authors:** Agnes Elmberger, Erik Björck, Juha Nieminen, Matilda Liljedahl, Klara Bolander Laksov

**Affiliations:** 1 Department of Learning, Informatics, Management and Ethics, Karolinska Institutet, Tomtebodavägen 18A, 171 77 Stockholm, Sweden; 2 Clinical Genetics, Karolinska University Laboratory, Karolinska University Hospital, Stockholm, Sweden; 3 Department of Molecular Medicine and Surgery, Karolinska Institutet, Stockholm, Sweden; 4 Primary Health Care Unit, Institute of Medicine, The Sahlgrenska Academy, University of Gothenburg, Gothenburg, Sweden; 5 Department of Education, Stockholm University, Stockholm, Sweden

**Keywords:** Activity theory, Change management, Clinical education, Educational change, Faculty development, Innovation, Knotworking, Knowledge transfer, Organisational change, Transformation

## Abstract

**Background:**

Faculty development is important for advancing teaching practice in health professions education. However, little is known regarding how faculty development outcomes are achieved and how change in practice may happen through these activities. In this study, we explored how clinical educators integrated educational innovations, developed within a faculty development programme, into their clinical workplaces. Thus, the study seeks to widen the understanding of how change following faculty development unfolds in clinical systems.

**Methods:**

The study was inspired by case study design and used a longitudinal faculty development programme as a case offering an opportunity to study how participants in faculty development work with change in practice. The study applied activity theory and its concept of activity systems in a thematic analysis of focus group interviews with 14 programme attendees. Participants represented two teaching hospitals, five clinical departments and five different health professions.

**Results:**

We present the activity systems involved in the integration process and the contradiction that arose between them as the innovations were introduced in the workplace. The findings depict how the faculty development participants and the clinicians teaching in the workplace interacted to overcome this contradiction through iterative processes of negotiating a mandate for change, reconceptualising the innovation in response to workplace reactions, and reconciliation as temporary equilibria between the systems.

**Conclusion:**

The study depicts the complexities of how educational change is brought about in the workplace after faculty development. Based on our findings and the activity theoretical concept of knotworking, we suggest that these complex processes may be understood as collaborative knotworking between faculty development participants and workplace staff through which both the output from faculty development and the workplace practices are transformed. Increasing our awareness of these intricate processes is important for enhancing our ability to make faculty development reach its full potential in bringing educational change in practice.

**Supplementary Information:**

The online version contains supplementary material available at 10.1186/s12909-020-02407-8.

## Background

Faculty development activities are key for supporting medical educators in their scholarly role as teachers [1, 2]. Despite a large body of literature exploring the outcomes of faculty development [2–4], there is still a gap in what we know about how these outcomes are achieved and how faculty development may contribute to change in educational practice [2, 5–8]. Here, we explore how clinical educators integrate educational innovations developed within a faculty development programme into their workplaces. This paper thus strives to contribute to the understanding of how change in relation to faculty development unfolds in clinical systems, and aims to inform future faculty development practices.

While there has been much research into the practices of faculty development, criticism has been levelled against the suitability of current research approaches [2, 5, 7, 9, 10]. Firstly, the extensive focus on the design and delivery of faculty development activities (“input”), or outcomes and effectiveness (“output”) (e.g. [2, 3, 11, 12]), has, of course, provided us with valuable insights. Yet, this research also tends to leave important questions about what happens between input and output; that is, *how* outcomes and change are achieved in the workplace [5, 7, 8]. There is research which, in part, addresses these questions by focusing on how faculty transfer knowledge and skills from faculty development [1, 13]. Although offering information on influencing factors such as personal characteristics, design of the program and workplace features [1, 6, 13], studies have been critiqued for failing to investigate how the identified factors interact, and thus provide little insight into the transfer or change process in itself [5, 7, 8]. Some studies explicitly focus on the practices of change in relation to faculty development training, recognising a number of challenges such as a lack of formal mandate, and the need to reach consensus with the workplace [14]. Also, studies have addressed the relationship between faculty development outcomes and mechanisms of change suggesting motivation, relationship building, and leadership support as some of the important mechanisms enabling, or when absent, limiting change [4, 7]. Nevertheless, despite these recent efforts, the burning question of how change in practice may happen following faculty development remains largely unanswered [2, 5, 7, 10].

Furthermore, many argue that current faculty development research does not fully recognise the context in which faculty work and where they will ultimately use what they learn, including how that context may influence outcomes from faculty development [2, 5, 9, 10]. This is a concern specifically expressed in sociocultural research where knowledge and learning is viewed as contextual and embedded in practice and where change is ultimately a process unfolding in the workplace [2, 7, 9, 10, 15]. In line with this perspective, more theoretically informed research focusing on the interplay between faculty and their workplaces is called for when studying transfer and change following faculty development activities [2, 5, 9, 10]. Adopting approaches sensitive to these interactions may be especially critical in health professions education, given the complexities of clinical workplaces in which tensions between clinical practice, research and education limit the opportunities for educational work and faculty development [16].

With the purpose of addressing some of the above mentioned criticism and to account for the complex nature of change as it unfolds in clinical systems, we apply activity theory in this study. Activity theory views human activity and knowledge as situated and highlights interactions between individuals and their social and cultural context [17]. The basic unit of analysis is one or several interconnected activity systems. In the system, a subject (a person or group, e.g. a physician) works towards an object (the aim of the activity) using tools (e.g. medical knowledge). Activity theory distinguishes between the specific object, present on an individual level in a given situation (e.g. diagnosing a particular patient), and the generalized object, present at a system level and connected to social meaning (e.g. patient care and disease prevention) [18]. Further, the subject is part of a community in which rules regulate the activity, and the division of labour specifies how power and tasks are divided within the community. Systems can come together in interaction and as they represent different perspectives and cultures, contradictions may arise between them. As individuals within the systems work to resolve the contradictions, the activity evolves, and as such, contradictions are seen as drivers of change.

In this study, we use a novel faculty development programme as outset. The programme, further described in the Methods section, aimed at supporting teams of clinical educators in developing innovations based in educational research and theory. The educational innovations are used as a point of departure to inform our understanding of how educational change in clinical workplaces may happen as a result of faculty development. Applying activity theory, the specific aim is to explore how clinical educators integrate educational innovations developed within a faculty development programme into their clinical workplaces.

## Methods

### Study design

The study was inspired by case study design [19] and focused on the faculty development programme as a case offering an opportunity to study educational change in relation to a faculty development activity. The study was explorative in nature and applied qualitative data collection methods to gain detailed accounts of participants’ experiences [20].

### Setting

This study invited participants from a one-year faculty development programme held in 2016 at a Swedish university offering several health professions education programmes. All heads of department at the university’s affiliated teaching hospitals received information about the programme, inviting teams of clinical educators (i.e. clinicians with specific educational roles including allotted time for education besides clinical work). A total of five teams enrolled, consisting of three to five participants. The programme aimed at supporting the teams in working with educational change by having each team develop and implement an educational innovation, which we in this paper define as a new educational intervention or tool. The innovations were to be informed by educational research and the overall aim should be to improve teaching practices. After identifying a need in their own setting, each team constructed specific aims for their respective innovations. For more information on the innovations, see Table 1.

**Table 1:** List of innovations developed in the faculty development programme

Innovation	Focus	Target group
Reorganisation of clinical rotation and a new learning activity^a^	Interprofessional communication and profession-specific tasks	Students
A new learning activity including an educational video resource	Interprofessional ward rounds	Students
An educational video resource	Student feedback	Teaching clinicians
Reorganisation of clinical rotation to create educational teams of two students and two teaching clinicians	Peer learning	Students and teaching clinicians
A new learning activity including an educational checklist	Interprofessional ward rounds	Students

^a^For further information, see [21]

**Fig. 1:**
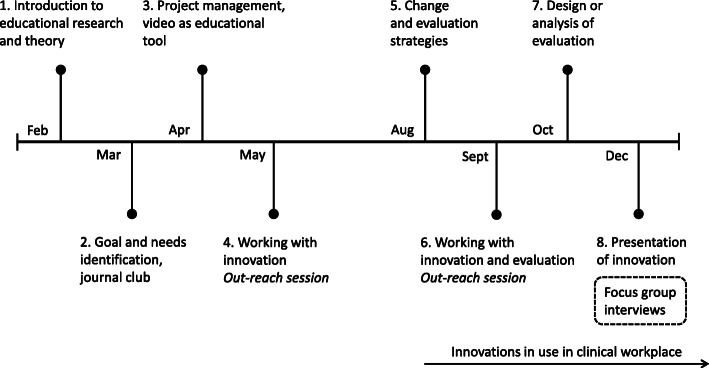
The faculty development programme

The programme included six half-day sessions at the university and two out-reach sessions in the workplace in which teams met separately with a programme facilitator. Each session had a specific focus, summarised in the timeline in Fig. 1, and time was allotted for working with the innovations during the sessions. By the end of the programme, each team had introduced their educational innovation in their workplace. For more information about the specific programme and how teams were supported in working with change, please see [22].

### Data collection

#### Participants

All programme attendees were invited to participate in the study by researcher AE. Two were unable to join, resulting in 14 study participants from two teaching hospitals and five clinical departments, all with both in- and outpatient practice. Internal medicine as well as surgical specialities were represented. Please see Table 2 for more information about participants.

**Table 2:** Participant characteristics (*N* = 14)

Category	
**Gender (nr)**	
Male	3
Female	11
**Age (years)**	
Mean	44
Range	30–63
**Profession (nr)**	
Physiotherapy	6
Medicine	4
Nursing	2
Occupational therapy	1
Social work	1
**Teaching experience (years)**	
> 5	8
3–5	4
1–2	2

#### Focus group interviews

We conducted focus group interviews to make use of the group dynamics that had been established during the programme. A semi-structured interview guide (Additional file 1) was created through discussions in the research group and included questions about the process of integrating the innovations and working with educational change in the workplace. The focus groups were conducted in conjunction with the last programme session (see Fig. 1). The focus groups, consisting of three to six participants, were organised so that teams were maintained: one group consisted of two teams working with similar patient categories, another group of one larger team, and a third group of two teams working with similar student groups. Each focus group interview was guided by a moderator (researchers AE, KBL, ML) who was assisted by an observer. The focus groups lasted around 1 h and were recorded. Informed consent was obtained from all participants before the interviews and the study was approved by the Regional Ethical Review Board in Stockholm (no: 2016/1425–31).

### Analysis

After verbatim transcription of the interviews, we performed a two-step theoretically driven analysis informed by activity theory. Specifically, we were guided by the concepts of interacting activity systems and contradictions as drivers of change [17] as we posed the following analytical questions: a) which activity systems were involved in the integration process and which were the contradictions between them? and, b) how could the interaction between these systems be understood?

To address the first question, four of the researchers (AE, EB, KBL, JN) individually read the transcripts and coded the data to identify activity systems and contradictions involved in the process of integrating the innovations in practice. We then compared our coding and through discussion reached agreement on the involved activity systems and the contradiction between them.

To address the second question, we used coding procedures from the thematic analysis approach [23]. Firstly, we re-read the transcripts and coded data pertinent to how participants worked with integrating their innovations (AE, EB, KBL). These codes were collated into initial sub-themes (all researchers). To this stage, the thematic analysis was performed without an activity theoretical perspective. Secondly, the set of sub-themes was subject to discussion in the research group and building on the activity systems we previously had identified; we now collated the sub-themes to construct themes that reflected the interaction taking place between the systems. To refine these emerging themes, the transcripts were revisited to perform another round of coding looking for any uncoded data before the final themes were named to reflect the underlying meaning of each theme. Lastly, quotes that reflected aspects of each theme were chosen and translated into English. NVivo (QSR International Pty Ltd. Version 11) was used to support the analysis. Examples of data extracts, codes, sub-themes and final themes are shown in Table 3.

**Table 3:** Examples of data extracts, codes, sub-themes and final themes

Data extract	Code	Sub-theme	Final theme
*Of course there are key stakeholders whom you need to be aware of and whom you need to get on board. (Focus group 3)*	Engage key stakeholders	Communication and brokering	Negotiating a mandate for change
*This [the innovation] has its own life in a way; the teaching clinicians […]* *change what we come up with. (FG2)*	Workplace using innovation different from intended	Workplace shaping its use of innovation	Reconceptualising the innovation
*If you’re implementing something on a large scale then everyone needs to be on board, everyone that’s affected, otherwise it won’t work. (FG2)*	Make sure everyone is on board	Reach agreement	Reconciliation between systems

### Trustworthiness

Researchers EB, KBL and ML were facilitators in the faculty development programme and we recognise that this might have impacted the study. However, the aim was not to evaluate the programme per se and AE and JN had no involvement in designing or teaching in the programme. Further, researchers first individually coded transcripts, followed by continuous discussions among all researchers to revise and review the interpretations and to challenge any assumptions not supported by the data.

## Findings

We begin by describing the activity systems involved in integrating the educational innovations and our analysis of the contradiction that arose between them. We then describe how the systems interacted in response to the contradiction.

### The contradiction: integrating a new innovation versus maintaining existing routines

**Fig. 2:**
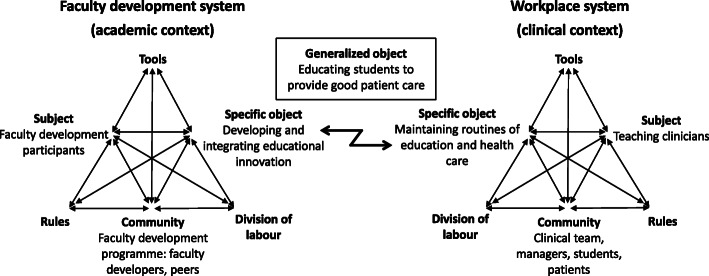
The involved activity systems. Integration of the innovation involved two activity systems sharing the generalized object. Zig-zag arrow marks a contradiction between the systems’ specific objects

The development and integration of the educational innovation involved two activity systems: *the faculty development system* and *the workplace system* (Fig. 2). The faculty development system held the programme participants, the team, as subjects, and the workplace system held those in the workplace who were involved in teaching as subjects, that is, the teaching clinicians. The programme participants were also part of the workplace system, as they too were teaching clinicians. However, in the specific activity of integrating the innovations, they were acting primarily as subjects in the faculty development system, and thus when referring to teaching clinicians, the programme participants are not included.

While the two systems shared the generalized object of educating students to provide good patient care, their specific objects appeared to be misaligned. The specific object of the programme participants was to develop and integrate the educational innovation into practice. However, the innovation constituted a new element in the workplace, requiring change of existing practices and routines. Thus, when the innovation was introduced in the workplace, the specific object of the teaching clinicians appeared to be to maintain functioning routines and practices of education and health care. Consequently, a contradiction arose between the specific objects of the two systems (zig-zag arrow in Fig. 2), with the team working to integrate their innovation, thereby requiring change, while the teaching clinicians worked to maintain routines and practices. This contradiction manifested as resistance among the teaching clinicians in the workplace. As one team noted:

*P2:5*^1^*: You were there [in the workplace] and acted as a sounding board and got to take all crap in the beginning.*

*P2:6: Oh yes.*

*P2:5: Which were more […] the general concern that now someone from outside comes and stirs it up on this ward that actually functions well […]*

*P2:6: And I never thought that I would say what managers usually say, when they [teaching clinicians] were attacking; ‘I hear what you say and I’ll think about it … ’ Really quickly it became apparent for me that there was no point in going into conflict. *

Further, the teaching clinicians questioned if they could, with the high workload, manage the change, and as this following excerpt illustrates, the team had to motivate the teaching clinicians to conduct the required changes:

*P3:2: Our innovation has, to a very high degree, required that our colleagues work in a different way with the students, they are doing the work really, we [the team] are just doing the planning so to speak. So they are highly involved and we’ve had to put in a lot of work to motivate them about this change and why it's good, [ …] and they did find it to be good but first they were sceptical as it required that they changed their way of working …*

*P3:5: Probably ‘oh, are we doing something new now … ’*

*P3:2: Exactly.*

*P3:5: It’s the thing with teaching old dogs new tricks; you'd rather just go on routines. *

### Interaction between the systems

Building on the two systems, we now describe how the systems and their actors interacted in response to the contradiction. In our analysis, we constructed three themes that reflect aspects of this iterative interaction, namely negotiation, reconceptualisation and reconciliation.

#### Negotiating a mandate for change

An important part of integrating the innovation seemed to involve negotiations of legitimacy and mandate to change existing workplace practices. The programme was discussed as facilitating this mandate and approval from managers to attend was described as a green-light to pursue the tasks involved in the programme. Further, as one participant noted, the programme situated the team in a bigger picture and anchored the innovation in educational research and expertise, thus empowering the team and adding legitimacy:

*This [the programme] became a mandate, [the innovation was] not only something we had come up with ourselves, but it actually had a foundation, […] so because of the setup, we acquired a mandate to carry through something. (P3:5)*

Moreover, the team structure seemed to contribute to the mandate for change, partly because it implied shared decision-making and ownership instead of being individually accountable for the innovation and the required changes. Participants also described how the team offered a broader network in the workplace where team members could create alliances with or involve different individuals in the project.

*We [the team members] know people in different ways, and we also have the other ward where I’ve worked, so I might have a more natural point of contact there. So I believe recognition is very important […] when someone brings something new, that people are able to say, ‘oh but I recognise this person and I can trust [him or her]’. (P3:4)*

Furthermore, the participants noted the importance of being perceived as in-group in the workplace as this contributed to a mandate to change and discussed that this could be achieved by having team members with the same professional belonging as the teaching clinicians. Also, having formal educational roles was described as providing authority to change educational practices in the workplace. However, it was evident that these formal roles alone were insufficient and that iterative negotiations between the systems were needed to acquire a mandate for change. Communication was recognised as an important component of this negotiation:

*It’s impossible to say who has the mandate; I have the formal mandate […], but it would be really stupid of me to just carry through something – that wouldn’t work, my name would show up to be quite weak, no one would do as I say, I would be declared a fool and people would hate me because there are so many that have mandate over the [education]. I’m not allowed to do something that messes things up for the others […] what’s required is that everyone finds it a good idea, […] so there have been quite a lot of conversations with a lot of people. (P2:5)*

The extent of communication varied between teams and participants described using both formal and informal communication channels, such as workplace meetings or chatting over coffee. Also, special efforts were made to communicate with key stakeholders such as managers or others responsible for the clinical education as these individuals were recognised as having an ability to influence the larger group of workplace staff.

*She [another team member] really made efforts to address the nurses […] and attend their meetings considering that they are in charge of the rounds, so they represent key individuals and they really have to adopt it [the innovation] for others to buy in too. (P1:3)*

#### Reconceptualising the innovation

The teams described how they had to consider alternative versions or conceptualisations of their innovations in response to resistance and questioning in the workplace. Thematised here as reconceptualisation, this involved iterative communication and interaction between team and workplace as it enabled the team to assess and respond to workplace reactions. The interaction could be more or less intentional; some teams actively engaged the workplace in providing feedback or taking part in developing the innovation. Also, participants emphasised the importance of communicating specifically with those who were affected the most by the innovation when assessing workplace reactions:

*There were reactions from the nurses, that it would be too much [work], that they didn’t have the capacity for that number of students. And then we realised, in spite of the positive reactions from management [to our original idea], the importance to anchor it [our innovation] at different levels. (P1:1)*

As such, reconceptualising the innovation included going back and forth between generating and testing ideas, assessing reactions in the workplace, and changing, adding or scaling down parts of the innovation in response to the reactions. Through this, the innovation was reconceptualised and adapted to better fit current workplace practices and reduce tensions within the workplace system, as described here:

*P2:6: Well, to begin with, all teaching clinicians … There were worries about how it would work in practice. Then there were lots of questions that we [the team] couldn’t answer, so we were actually forced to think them over, [ …] and that’s where I said ‘just take it easy, this will be fine, we will just think it over, we will get back [to you]’ [laughing].*

*P2:5: Exactly, it was the worry … they focused on the details […] And you were there [in the workplace], acting as a sounding board and then we took some of the reactions into account, we toned down some things and made other things more specific…*

*P2:4: And then you also wrote some good instructions for the teaching clinicians which I believe eased the pressure a bit …*

Further, the reconceptualisation appeared to extend beyond adaptations conducted by the team to also include how the workplace shaped its use of the innovation. Participants described how the teaching clinicians deviated from the procedure originally intended by the team and used the innovation in ways different from those instructed. One team noted:

*P2:5: This [the innovation] has its own life in a way; the teaching clinicians […] change what we come up with.*

*P2:4: And they do it differently too.*

*P2:5: It took like one and a half weeks until they changed fundamental things that I didn’t [plan], but I haven’t said anything because that’s just fine, it’ll be fine.*

*I: What do they change?*

*P2:4: Well there’s been some things, like they didn’t think, in the beginning I wanted us to use simulated cases but then ‘I didn’t want to and I changed this, I did something else’, so […] either they do it differently or they make up their own cases …*

#### Reconciliation between systems

At the end of the programme, all innovations were in use in the workplace. As such, the interaction between the systems had achieved varying degrees of change in workplace routines and practices, primarily related to the extent of change required by the specific innovation.

*The feedback we’ve received so far has been generally positive and we’ve also received very spontaneous feedback from other professions who’ve reacted positively on the new way of working, so I think that says something, when people in the periphery also notices that there’s some difference […] In that sense, it feels like a successful project and the tendency seems to be that we will achieve some of the goals that we initially had … (P3:2)*

This phase, which we name reconciliation, implied that the systems had reconciled their initially misaligned objects through negotiation and reconceptualisation, and agreed on working according to the structure implied by the innovation:

*P2:6: Occasionally it’s been really tense; in the beginning they [teaching clinicians] said ‘this will never work’, ‘yes it will’, ‘no’, I remember that.*

*P2:5: Who won then?*

*P2:6: We won because we reached agreement [with the teaching clinicians].*

However, the reconciliation did not appear as a solid endpoint, but rather as a temporary phase of equilibrium between the systems. The participants described that the innovation was continuously subject to re-negotiation and change in response to new tensions, which could arise as new activity systems or actors, such as students, encountered the new practice. Hence, the reconciliation seemed to be part of the iterative interaction, with fluidity between negotiation, reconceptualisation and reconciliation.

*P2:4: We’ve had several [students] this semester who’ve been really afraid and felt very vulnerable and felt a responsibility [when working according to the new structure] […]*

*P2:6: It has brought something to life, we can’t just stir the pot and then leave what’s happening behind, we really need to attend to this.*

## Discussion

In this study, we set out to contribute to the understanding of change following faculty development by investigating how educational innovations developed within a longitudinal faculty development programme were integrated into clinical workplaces. Applying activity theory, we identified two activity systems involved in the process: the faculty development system and the workplace system, between which a contradiction arose as their objects were not fully aligned. The findings further illustrate how the systems came together in interaction including iterative processes conceptualised as negotiation, reconceptualisation and reconciliation. Our findings on the critical issue of negotiating mandate for change by relying on social networks; using leverage provided by the faculty development programme and formal educational roles; and taking advantage of bringing change from within, aligns well with previous research on how faculty development participants work with change in practice [4, 7, 24]. Further, as others also have identified [6, 7, 24], communication with the workplace community was an important aspect in incorporating the innovation and bringing about change. However, what the current study particularly adds lies in the emphasis on the interactive, fluid and ongoing nature of incorporating the innovations in practice. In the following paragraphs, we draw particularly on the concept of knotworking [25] to illustrate this complex interaction between systems and discuss what the findings can tell us about change in relation to faculty development.

**Fig. 3:**
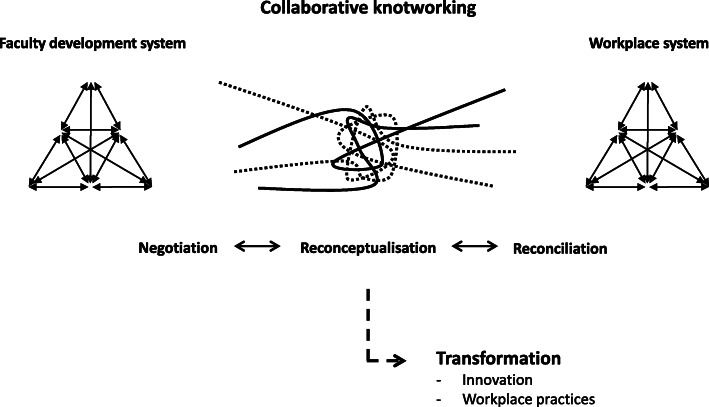
Collaborative knotworking between the faculty development system and the workplace system. The activity systems interacted through knotworking in response to the contradiction. Both systems contributed with threads and the knot was continuously untied and retied (represented with dashed threads). Through this collaborative knotworking, which included iterative processes of negotiation, reconceptualisation and reconciliation, the innovation and the workplace practices were transformed

Knotworking offers a way to describe what happens when activity systems or organisations come together in interaction and collaboration [17, 25, 26]. The site for the interaction is illustrated as a knot and the knotworking itself can be understood as a separate kind of activity that forms between the systems. In this study, the faculty development system and the workplace system were brought closer together in such a knot when the team worked towards their object of integrating the innovation into the workplace (Fig. 3). The systems and their actors, practices and elements each constituted a thread in this knot, and the threads sometimes pulled in different directions due to the contradiction between the systems. In response to this contradiction, the two systems and the individuals within them engaged in collaborative knotworking including processes of negotiation, reconceptualisation and reconciliation. The *negotiation* served to mediate and align the different practices and objects of the two systems, which also required *reconceptualising* the innovation. Knotworking is further characterised by ‘pulsations’ in which the knot is tightened and loosened, untied and retied (illustrated with dashed threads in Fig. 3) [25]. This relates to the iterative interaction found between the systems in this study, where the *reconciliation* between team and workplace constituted a temporary phase of equilibrium rather than an endpoint. As such, the concept of knotworking further emphasises the complex and collaborative nature of faculty development workings and outcomes, and provides a useful metaphor which, coupled with the findings of this study, raises some interesting reflections about how faculty development may contribute to change in practice.

First, this study does not illustrate the innovations created within the programme as decontextualised and fixed objects to be transferred to existing practices. Instead, the perspectives of the two systems were negotiated in the knot whereby the innovations were subject to questioning, reanalysis and reconceptualisation. Supported by previous research, this indicates that educational change is inherently local [15, 24] and that innovations are transformed and reconceptualised in relation to the specific workplace as they are being integrated [27, 28]. Interestingly, although the innovations in this study constitute rather tangible outputs, our findings are consistent with research focusing on more traditional, less tangible, faculty development outputs such as knowledge and skills. Studies show that that these outputs are indeed contextually bound, filtered and interpreted in relation to the structures and practices within the workplace [15, 24, 27]. This is also in line with the conceptual argument that knowledge and skills are not fixed entities to be acquired in one context (e.g. a faculty development programme) and transferred to another (e.g. the clinical workplace) [10, 29, 30]. Drawing upon our findings in this study, we are highly inclined to agree: outcomes following faculty development are achieved not through linear transfer but rather through an act of collaborative knotworking where outputs are transformed as they are integrated in practice.

Second, this study offers some insights into how existing workplace practices and routines were negotiated through the collaborative knotworking and that integrating the innovations required change in these practices. As such, the knotworking also served to motivate staff to change their previous ways of working. This is in line with the results of Kerosuo and Engeström [27], who found that integrating new innovations in healthcare triggered a reshaping of routines and activities. It further relates to one of the integral notions of activity systems, namely that all elements are interconnected [17] and that new elements will collide with existing practices and rules when they are introduced in a system, thus creating tensions and requiring the system to transform [26, 27]. In other words, the findings of this study not only suggest that outputs from faculty development are transformed as they are integrated in the workplace, but also that current workplace practices are transformed as a result of that integration.

Third, although the workplace is well known as an important factor influencing outcomes from faculty development [6, 7, 13], our findings complement this perspective by depicting it as a participating actor. From resistance and questioning, through negotiation and reconceptualisation, the workplace expressed agency and influence in the process where each teaching clinician had the power and choice to shape their use of the innovation. As such, the findings highlight how both team and workplace were agentic, which corresponds well with what Engeström [25] argues: that knotworking is not reducible to a single centre of control but rather is a collective act in which the initiative continuously changes between individuals or organisations. From this perspective, and as others too have argued [5, 7, 10, 29, 30], faculty development programmes and the workplace are not isolated and separate systems, and programmes cannot contribute to change in the workplace simply through a linear and unidirectional transfer between these contexts. Rather, we here suggest the concept of collaborative knotworking to reflect the interaction taking place between faculty development participants and their workplaces, transforming both the programme outputs and workplace practices.

### Considerations

This study invited participants from a programme focusing particularly on innovations, and while this differs from typical faculty development outputs such as knowledge and skills, the study generated several findings which, potentially, can inform other situations and contexts. Further, the study is based on the perspectives of the programme participants, while it would have been valuable to also include other stakeholders such as workplace staff. As such, more research is needed to further elaborate on the concepts of knotworking and transformation and investigate them in relation to different kinds of faculty development efforts. Also, while the use of activity theory offered insights into interactive processes between systems, it might have disregarded other significant aspects, such as individual change agency, which could be the focus of future studies.

**Fig. 4:**
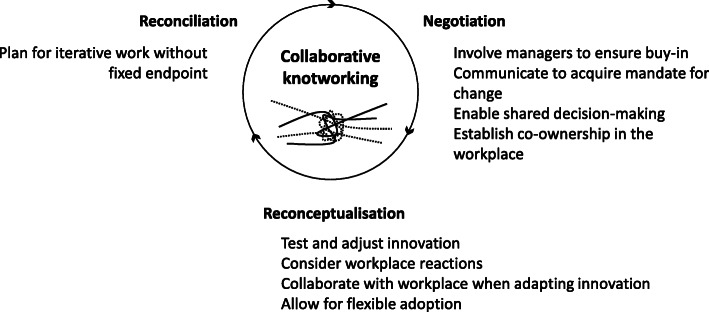
Issues to consider when navigating through negotiation, reconceptualization and reconciliation

Moreover, as the programme was voluntary, the participants’ experiences might differ from those who engage in faculty development for other reasons. However, we believe that the findings may reflect the experiences of many ‘different’ participants in its emphasis on transformation and interaction between systems. Further, the study participants represented different professions as well as several clinical workplaces in two teaching hospitals. The findings could thus possibly be relevant for a wide range of healthcare professionals. Finally, although data for this study was collected in 2016, we believe our findings can still offer useful guidance and insights for faculty developers as well as faculty development participants in how to work with educational change and knotworking in clinical contexts. For a summary of how clinicians may navigate through this knotworking and the three involved aspects, see Fig. 4.

## Conclusions

For faculty development to reach its full potential in bringing about educational change, we must increase our understanding of how such change unfolds in clinical workplaces and how participants go about applying what they learn in practice. Focusing on innovations developed in a faculty development programme, this study suggest that outcomes and change in practice are achieved through collaborative knotworking between the faculty development programme, its participants and their workplaces, which transforms both the outputs of the programme and the current workplace practices and activities.

We must not expect participants to navigate this complex process on their own, but should offer support and guidance as part of faculty development. This necessitates that we acknowledge the context in which participants work and explicitly address knotworking and transformation in our programmes. As participants return to their workplaces, they must recognise that their knowledge or innovation will transform through iterative and collaborative knotworking with the workplace, going back and forth between negotiation, reconceptualisation and reconciliation. Only when we recognise the complexities of these processes can we work to facilitate them, thus making sure faculty development reaches its full potential in contributing to educational change in practice.

## Supplementary Information

**Additional file 1.** Semi-structured interview guide.

## Data Availability

The datasets generated and analysed during the current study are not publicly available due to the risk of participants being identified but are available from the corresponding author on reasonable request.

## Footnotes

1 First number (here 2) indicates focus group, second number (here 5) indicates code assigned to participant during transcription.
