# Supplementary material for: Collaborative knotworking – transforming clinical teaching practice through faculty development
Source: BMC Med Educ. 2020 Dec 9;20:497. doi: 10.1186/s12909-020-02407-8 (PMC7726860; doi:10.1186/s12909-020-02407-8)
Supplement: Supplementary file 1 — Additional file 1. Semi-structured interview guide. [file 12909_2020_2407_MOESM1_ESM.pdf]

Additional file 1: semi structured interview guide for focus group interviews

Introduction

- Overview of study aim
- Provide information about informed consent including reminder that all participation is voluntary and may be cancelled at any time without having to state why
- Inform about the purpose of the observer + recording
- Inform about rules for discussion:
    - An open discussion
    - Talk one at a time, and avoid interrupting each other
    - Aim for everyone to participate actively in the discussion

---

This guide only includes main themes and questions to be discussed in the group. The interviews are semi-structured, allowing for exploration of new questions and discussions arising during the interview. Also, general prompts may be used and are not included in the guide (e.g. tell me more about that, can you describe what you mean by that?).

Starting point

• Can you tell us about the project's integration and results?

Resources and space to work with the project

• What space and resources have you had to run your projects?
  To be able to take part in this programme?
  Have you had time set aside to work with the projects?
  How was this space created? Has anyone given you this space - if so, who?
  Have you been given formal educational roles with time for educational tasks? How did that happen?

The workplace context

• Can you describe the process you used to inform about your projects in the workplace?
  How did colleagues and managers learn about what you are doing?
  In what contexts (forums, meetings, information sheets, e-mails) were the projects discussed or mentioned?
• How have your workplace and colleagues received or reacted on the project?
• Can you tell us about the process taking place in your workplace to integrate the project?
  Have your colleagues been involved? In what way/how?
  Which individuals were involved in your work? Why and how?
  What was difficult? Why? How did you work with that?
• What is it that keeps other colleagues from getting involved in the same way as you?
• Are there any “de-motivators” or difficulties when it comes to educational engagement? Can you tell us more about them?

Mandate and influence

• Can you tell us about your experiences in terms of mandate for change?

Who has the mandate to change? Who must be involved? (management, colleagues (who?), yourself)

• What mandate do you have - both formally (roles at the workplace?) and informally?
• What are your opportunities for influence at your workplace?

Team and programme

• Can you describe the development in the team, how did that process unfold?
• What has it meant to do this project in teams instead of individually?
• Can you tell us about the work in the team during the integration phase - who has done what, why?
• What role has the program had for your projects?

Finish

• If you were to do something similar again - what would you do differently and what would you do the same? What have you learned in terms of working with change?
